# The politics behind the implementation of the WTO Paragraph 6 Decision in Canada to increase global drug access

**DOI:** 10.1186/1744-8603-8-7

**Published:** 2012-04-03

**Authors:** Laura C Esmail, Jillian Clare Kohler

**Affiliations:** 1 Leslie Dan Faculty of Pharmacy, 144 College St., Toronto, ON, Canada

**Keywords:** Access to medicines, Drug access, Pharmaceuticals, HIV/AIDS, Intellectual property rights, Patents, Compulsory licensing, TRIPS, Paragraph 6, Human rights, Canada's access to medicines regime

## Abstract

**Background:**

The reform of pharmaceutical policy can often involve trade-offs between competing social and commercial goals. Canada's Access to Medicines Regime (CAMR), a legislative amendment that permits compulsory licensing for the production and export of medicines to developing countries, aimed to reconcile these goals. Since it was passed in 2004, only two orders of antiretroviral drugs, enough for 21,000 HIV/AIDS patients in Rwanda have been exported. Future use of the regime appears unlikely. This research aimed to examine the politics of CAMR.

**Methods:**

Parliamentary Committee hearing transcripts from CAMR's legislative development (2004) and legislative review (2007) were analysed using a content analysis technique to identify how stakeholders who participated in the debates framed the issues. These findings were subsequently analysed using a framework of framing, institutions and interests to determine how these three dimensions shaped CAMR.

**Results:**

In 2004, policy debates in Canada were dominated by two themes: intellectual property rights and the TRIPS Agreement. The right to medicines as a basic human right and CAMR's potential impact on innovation were hardly discussed. With the Departments of Industry Canada and International Trade as the lead institutions, the goals of protecting intellectual property and ensuring good trade relations with the United States appear to have taken priority over encouraging generic competition to achieve drug affordability. The result was a more limited interpretation of patent flexibilities under the WTO Paragraph 6 Decision. The most striking finding is the minimal discussion over the potential barriers developing country beneficiaries might face when attempting to use compulsory licensing, including their reluctance to use TRIPS flexibilities, their desire to pursue technological development and the constraints inherent in the WTO Paragraph 6 Decision. Instead, these issues were raised in 2007, which can be partly accounted for by experience in implementing the legislation and hence a greater representation of the interests of potential beneficiary country governments.

**Conclusions:**

The Canadian Government designed CAMR as a last resort measure. Increased input from the developing country beneficiaries and shifting to institutions where the right to health gets prioritized may lead to policies that better achieves affordable drug access.

## Background

Global inequity in access to medicines continues to persist despite an increase in international efforts to address this problem over the last decade. The WHO estimates that approximately two billion people still lack regular access to essential medicines, which can be defined as the "equitable availability and affordability of essential medicines" [1-3]. For example, approximately 9.8 million of an estimated 15 million people living with HIV/AIDS in low and middle-income countries remain without access to antiretroviral therapy [4]. The emergence of new global actors such as the Gates Foundation and the Global Fund to Fight AIDS, Tuberculosis and Malaria has helped but not enough.^a ^The inequity in drug access extends to chronic diseases as well, with one study finding access levels of 7.5% to medicines for the treatment of cardiovascular disease, diabetes, chronic respiratory disease, glaucoma and palliative cancer care in six low- and middle-income countries [5].

There are many factors that contribute to these drug access inequities. The cost of drugs is a major obstacle, due to either the poverty of individuals and governments or high prices on the supply side [2]. Health systems infrastructure and its capacity to serve its population is a major obstacle, with inadequate facilities and lack of trained health professionals making it difficult to treat patients. Corruption in the pharmaceutical sector is a problem as well diverting scarce resources away from health systems [6]. Inappropriate drug use is a problem, which includes inappropriate prescribing by health professionals, inappropriate medicine use, and poor compliance by the end user [2]. These are only some of the barriers to drug access and as such, it is not a 'single failure' problem [2]. The lack of drug access is often the result of a combination of failures in the market, in government and even among aid players.

Crafting policy in this area is challenging precisely because drug access is a multi-faceted and complex issue. Just as there are many ways to think about and define drug access, there are many ways to address the problem of getting the right drug to the right person at the right time, and from a political perspective, every solution favours some groups more than others. With vested interests at stake, how drug access and its potential solutions are framed is a highly charged political exercise.

This paper examines the influence of framing, institutions and interests on the development of a compulsory licensing policy, which aims to increase access to medicines in developing countries. The case study under examination is Canada's Access to Medicines Regime (CAMR) originally known as the Jean Chrétien Pledge to Africa, which implements the WTO Paragraph 6 Decision [7]. To date, CAMR is the only example of the use of the WTO Paragraph 6 Decision internationally. In theory, CAMR allows Canadian generic manufacturers to produce patented medicines under a government-issued compulsory license for export to developing countries; [8] however, the bill is considered by many as an inadequate method to encourage improved drug access [9-11]. Since it was passed in 2004, it took three years for a group of civil society advocates and one Canadian generic company, Apotex, to produce and export a limited drug supply under CAMR. These efforts resulted in one order of antiretroviral tablets, enough to treat 21,000 HIV/AIDS patients in Rwanda for one year [11]^b^. Since then, representatives of developing countries and generic manufacturers have stated that the regime is too cumbersome and difficult to use and will unlikely use it again unless it is reformed [11].

### Research objectives

This research aims to examine the politics underpinning CAMR, by evaluating how stakeholders framed access to medicines and how framing, institutions and interests interacted and influenced CAMR.

### Theoretical framework

To answer these questions, a conceptual framework of framing, interests and institutions was used. Frames are defined as "a policy position resting on underlying structures of belief, perception and appreciation" [12]. In conjunction with framing theory, this research uses Stone's four-value framework to analyse actors' discourse during the policy debates, which posits that political struggles typically revolve around four main abstract policy goals: equity, efficiency, security and liberty [13]^c^. Interests are defined as the preferences of actors, which depend upon the potential effects of a policy [13]. Institutions are defined as "recognized patterns of behaviour or practice around which expectations converge" [14]. Institutions refer to government structures and agencies, as well as the formal and informal rules that exist internationally and domestically. Related to institutions, but cutting across the entire theoretical framework, is Scope of Conflict theory, which argues that institutions can affect how a problem is defined in the first place, who gets involved in a policy debate, and what arguments get more weight than others [15].

In order to reach a solution, an issue must be defined to some extent, a process called framing; however, every definition and simplification emphasizes some aspects of the problem at the expense of others [12]. The analysis of stakeholder framing can expose what policy options were ruled out from the beginning, what policy options were actually considered, what issues or arguments stakeholders and governments failed to consider in a policy debate, and finally, how all of these factors influenced the outcome of the policy debate.

Moreover, different institutions value these frames and interests differently and public policy literature suggests institutions are a key determining factor in how the problem and the alternatives available are framed and which ones matter more than others [13,15,16]. Institutional structures and processes influence how a problem is defined in the first place, which defines what is at stake, the interested parties, and what ideas get privileged over others. Altogether, institutions, framing, and interests influence the trade-offs that participants in the policy conflict will have to make, which ultimately restricts the policy outcome.

### TRIPS, the Doha Declaration and the Paragraph 6 Decision

The Trade-Related Aspects of Intellectual Property Rights (TRIPS) Agreement outlines the minimum standards of all WTO Member countries for the protection of intellectual property rights [17]. In return, countries could pursue their goals of a strong domestic economy through access to global markets and increased technology transfer to least-developed countries. These policy goals took their roots in the principles of the efficiency of free, unfettered markets. Pursuant to TRIPS, patent holders are given a limited set of rights that includes exclusive marketing rights for a set duration of time. TRIPS obligations include 20 years of patent protection from the inventor's filing date (Article 33), patent rights free of discrimination against the origin of invention or production (Article 27.1), and exclusive marketing rights for the entire patent duration (Article 28) [18].

TRIPS grants limited flexibility around patent rights to Member countries to address public health needs.^d ^Article 31, otherwise known as "compulsory licensing", permits a government to issue a third-party license without the patent-holders' consent.

The premise underlying compulsory licensing is the price advantage of generic competition.^e ^Médecins Sans Frontières reports that the prices of antiretroviral therapy in the developing world decreased to the lowest unprecedented levels as a result of global generic competition, particularly coming from Indian manufacturers [19]^f^. Furthermore, nothing restricts the application of TRIPS Article 31 to chronic diseases [20].

The Doha Declaration, unanimously passed by WTO members in November 2001, clearly states that "[t]he TRIPS Agreement does not and should not prevent Members from taking measures to protect public health...we affirm that the Agreement can and should be interpreted and implemented in a manner supportive of WTO Members' right to protect public health and, in particular, to promote access to medicines for all" [21]. This suggests that countries should be able to make use of compulsory licensing and other flexibilities as they see fit. But, compulsory licensing is only worthwhile if manufacturing capabilities exist.

Two years later, the Paragraph 6 Decision was passed, which allows countries with manufacturing capacity to produce and export generic versions of patented medicines under compulsory license to countries without sufficient pharmaceutical manufacturing capacity.

### Canada's policy legacy in pharmaceuticals and intellectual property

Canada has a strong history of using compulsory licensing towards the production and importation of pharmaceuticals. In 1969, the Liberal Government amended its Patent Law to better facilitate compulsory licensing, after a series of reports identified patent protection as one of the major contributors to high drug costs in Canada [22]^g^. Canada's generic pharmaceutical manufacturing industry grew as a result of widespread compulsory licensing of pharmaceutical products [23]. From 1969 to 1992, Canada issued 613 licenses import or manufacture medicines under compulsory license [24]. The Government did not believe Canada was losing economically from it, and given Canada's negligible role in pharmaceutical innovation, the government did not see compulsory licensing making any impact on the R&D budgets of the innovative pharmaceutical industry. From the government's perspective, the policy decision traded away only the profits of the patent-holding companies [25].

The story changed significantly in 1987, when Prime Minister Brian Mulroney signed the Free Trade Agreement (FTA) with the United States [22]. Bill C-22, which amended the Patent Act and extended patent protection to 17 years in Canada, is viewed by some as linked to Canada's accession to the Free Trade Agreement with the United States and heavy lobbying by the multinational pharmaceutical industry [26]. The Mulroney government justified the change in patent law on the three basic premises: it reconciled domestic regulation with internationally mandated standards; it boosted R&D in Canada; and it incorporated Canada as a contributor to global pharmaceutical R&D [25]. From a domestic economic standpoint, the government wanted to steer Canada towards a more competitive, "knowledge-based economy" and in return, it would receive access to more open U.S. markets [25]. Bill C-91, introduced in 1993, was required for Canada's accession to the North American Free Trade Agreement (NAFTA) and the World Trade Organization (WTO) [25]. It effectively abolished compulsory licensing from Canada's mechanisms to contain escalating drug costs. Overall, Canada's policy shift in the area of pharmaceutical intellectual property protection took place in a context of a global movement towards increasing standards of intellectual property protection and the promotion of the idea of building competitive, knowledge-based economies.

### Introduction of CAMR

Canada's Access to Medicines Regime (CAMR) implements the WTO Decision on the Implementation of the Paragraph 6 Decision of the TRIPS Agreement into Canadian domestic law [7]. It amended the Patent Act and Food and Drugs Act to allow the limited use of government-issued, third-party licensing in Canada for humanitarian purposes. Specifically, it permits Canadian generic drug manufacturers to produce a "lower-cost version of a patented drug or medical device for export to developing countries that do not have the capacity to manufacture such products"[8].

After intense lobbying by Canadian civil society and a call to the Canadian federal government by Stephen Lewis, then UN Special Envoy for HIV/AIDS in Africa, the Canadian Government under then Prime Minister Jean Chrétien announced it would amend its Patent Act and implement the WTO Paragraph 6 Decision and on November 6, 2003, the Liberal government introduced Bill C-56^h^, An Act to Amend the Patent Act and the Food and Drugs Act [27]^i^.

### How CAMR works

CAMR's process and pitfalls are outlined in detail elsewhere, but the following provides a brief overview [28-30]. In order for a compulsory license to be issued, and a drug shipment sent to a country, several preliminary steps and criteria must first be met: 1) the importing country's eligibility must first be confirmed, as to whether it falls in one of three lists; 2) the medicine must first be deemed eligible as indicated by a schedule; 3) the generic company must first make efforts to obtain a voluntary license from the patent holder, of which there can be multiple; and 4) the generic company must first enter a contract with an importing country, without holding the license to produce the product. Through a procedure under the Governor in Council, countries can request the addition of a medicine or product. NGOs can use the regime to distribute medicines to another country only if they obtain an authorization from the importing country government.

Upon receiving the compulsory license the pharmaceutical company must meet all anti-diversion requirements, determine the royalty payment due to the patent holder, and establish a website with up-to-date details regarding the shipment.^j ^A compulsory license is valid for two years with a one-time two-year renewal if the specified quantity has not yet been shipped. Patent-holders can challenge a license if they believe it is being used for "commercial purposes"^k ^or if diversion of the medicines occurs, which then leads to the termination of the license^l^.

Since the passage of the legislation, only Rwanda has used the regime once to receive two shipments of antiretrovirals -- enough for 21,000 HIV/AIDS patients in Rwanda. This required enormous effort on the part of an alliance of NGOs and a Canadian generic company- Apotex. Since then, Apotex has publicly stated that it is reluctant to participate in the initiative again unless changes are made to streamline the regime [31].

The Liberal Government's announcement of reintroducing compulsory licensing to assist developing countries took many by surprise. While the policy move received some praise primarily in the domestic press, once the policy details started to surface, both civil society activists and the generic industry started to doubt the Government's intentions. What started as a beacon of hope for the international community, soon turned out to be a highly complex policy - a last resort measure. Despite attempts to review the policy and amend it, the regime remains as it is today: a public policy failure.

### Policy development and review process

The regime underwent two deliberative and developmental processes from 2004 through 2007: 1) in 2004, which sought public consultation with stakeholders to help inform the development of the policy, and 2) in 2007, which sought public input from stakeholders to review the regime and potentially amend it. After this consultation, the Canadian Government tabled its report to Parliament and concluded that the case for making legislative or regulatory changes to CAMR had not yet been made [32].

## Methods

This is a single case study, which used a comprehensive sampling strategy within specifically designated data boundaries. Data collection focused on two hearings of the Parliamentary Standing Committee on Industry, Science and Technology regarding CAMR. The first set of hearings consulted stakeholders on CAMR's draft legislation known as *Bill C-9, An Act to Amend the Patent Act and Food and Drugs Act*. These hearings took place from February 24, 2004 to April 22, 2004. The second set of hearings reviewed the implementation and outcomes of CAMR from April 16, 2007 to April 23, 2007. The hearings were open to the public and witnesses were selected by the Parliamentary Standing Committee based upon several criteria, including the type of study and the amount of time available. These are publicly available and well-defined data sources that informed who took part in the hearings (actors) and what they said (frames) leading up to the formation of the legislation and during the mandatory legislative review.

A content analysis was used to analyse the frames that actors expressed through the government consultation processes. This study adopted a mixed method approach to the content analysis, which involved two distinct stages and two distinct assumptions: first, a quantitative content analysis was performed assuming positivist approach;[33] second, the theme contents were summarized assuming a critical realist approach [34]. First, quantitative content analysis was chosen because it allows for a more explicit view of patterns that lead to the associations and conclusions. Counting the codes permitted the comparison of who said what on a macro-level: the level of emphasis that stakeholders placed on various issues was one indicator of what their key interests were in this policy debate. This quantitative content analysis produced categorized sections of text according to theme and stakeholder. The textual contents of these categorized sections were then summarized qualitatively. These summaries provided context to the quantitative results, which then provided a second level of detail on stakeholder interests and more comprehensively answered the question of how stakeholders framed access to medicines in the case of CAMR. Furthermore, the political context obtained through the qualitative summaries provided an important backdrop by which to understand specifically what factors ultimately shaped Canada's implementation of the Paragraph 6 Decision.

The creation and development of a codebook involved the following steps: 1) consultation of the literature to develop an initial list of themes and corresponding keywords, 2) application of the initial codebook to samples of data, 3) adjusting themes and keywords based upon these initial results (to be described below), and then 4) proceeding by coding the entire set of documents. To see the codebook, please consult Additional file 1.

To ensure reliability two researchers coded sample documents until an inter-rater reliability of 80% was achieved. Eighty percent inter-rater reliability is deemed acceptable when manually coding [33,35]. Subsequently, one researcher (LE) coded the full set of documents using text-analysis software, NVivo,^m ^and reviewed all text coded to ensure consistency of coding. To ensure little to no content was missed, all transcripts were searched for keywords and phrases using the text query function in NVivo, and then manually coded if deemed relevant. The unit of analysis is a complete session of hearings, which in the case of 2007, includes 3 hearings.

## Results

In 2004, a range of witnesses testified before the Standing Committee, as shown in Table 1. The stakeholder groups listed represent all of the witnesses who testified. The criteria used to categorize witnesses into stakeholder groups were group membership (for institutional interest groups) or their historical affiliation with other groups and their policy positions (for issue-oriented interest groups). Government representatives fell into three major categories: 1) the ruling government, which includes cabinet members and civil servants and 2) Members of Parliament (MPs) on the standing committee, which included 8 MPs representing the Liberal party, 4 representing the Conservative Party of Canada (CPC), 2 representing the Bloc Quebecois (BQ), and 1 representing the New Democratic Party (NDP), and a Chair from the Liberal party.^n ^Civil society consisted mostly of non-profit aid organizations and few academics who often advocate alongside these groups. The research-based and generic pharmaceutical industries consisted of representatives of their membership-based groups and company executives.^o^

**Table 1:** Standing committee witnesses in 2004 and 2007

						Members of Parliament				
						
	Government Officials	Research- Based Industry	Generic Industry	Civil Society	Intellectual Property Institute of Canada	Bloc Quebecois	Conservatives	Liberals	New Democrats	Independent
2004	7	2	2	18	2	2	4	9	1	0
2007	5	3	2	5	0	2	4	4	1	0

In 2007, a smaller scope of actors testified before the Standing Committee; however, a much larger scope of actors, from international and domestic organizations, submitted written consultations compared with the 2004 debates. The legislative review was well-publicized and facilitated through a website where the government posted a consultation paper listing specific questions regarding the regime. Any public individual or group was invited to make a written submission in response to the consultation paper.

For the 2007 review, consultations were held over three days between April 16, 2007 and April 23, 2007. Civil servants from the Departments of Industry Canada, Health Canada, Foreign Affairs and International Trade and the Canadian International Development Agency testified on April 16, 2007. Representatives from civil society organizations testified on April 18, 2007 and representatives from both generic and research-based industries testified on April 23, 2007.

Tables 2 through 5 present the results describing frequency with certain themes and issues were discussed in both 2004 and 2007. This quantitative presentation provides an overview of the policy goals and themes that drove the debate.

**Table 2:** Proportion of debates coded by policy goal^1, 2, 3^

	Equity		Security		Liberty		Efficiency	
				
	n	%	n	%	n	%	n	%
2004	37187	34	20642	19	51497	47	24921	23
2007	15964	29	15213	27	20777	37	19421	35

^1 ^For 2004 debates, the denominator = 108536 words (i.e. total number of words in ALL 2004 debates)

^2 ^For 2007 debates, the denominator = 55530 words (i.e. total number of words in ALL 2007 debates)

^3 ^These percentages aggregate all stakeholders and political parties. Percentages represent proportion of text where specified themes were found. Paragraphs often made reference to more than one concept; therefore paragraphs were often coded for multiple themes.

The frequency of themes in 2004 (Table 2) illustrate that the debates were primarily focused on issues related to liberty (47%) and equity (34%). More specifically, Table 3 illustrates that the participants in the 2004 debates appeared to be mainly arguing over: 1) where to draw the line with respect to protecting intellectual property (31.8%); and, 2) how to interpret the various clauses of the WTO TRIPS Agreement (18.5%). Participants' focus on equity (34%) was mainly about: 1) what the benefits of the regime should entail (List of Medicines, 12.4%); 2) who would be eligible for these benefits (10.6%); and, 3) the role of aid in increasing access to medicines (11.5%). Issues that were less frequently discussed related to efficiency (23%) and security (19%). More specifically, these under-represented issues included market competition (5.1%), procurement practices and protocols (3.1%), litigation (2.5%), innovation (2.0%), human rights (1.4%) and Canadian domestic economic issues (0.1%). To the extent that these issues were deemphasized, they were not what participants argued about during the debates. Tables 4 and 5 illustrate the breakdown of themes by stakeholder group and political party for the 2004 and 2007 deliberations.

**Table 3:** 2004 versus 2007 frequency of themes

	**2004**	**2007**
		
**Liberty**		
Intellectual Property	31.8%	27.8%
WTO or TRIPS	18.5%	14.5%
Right of Refusal	10.7%	0.0%
Developing Country Pressure	0.5%	2.0%
**Equity**		
List of Medicines	12.4%	5.0%
Aid	11.5%	23.1%
List of Countries	10.6%	1.7%
Equal Opportunity to Supply	2.1%	0.3%
**Efficiency**		
Diversion	6.1%	2.5%
Market Competition	5.1%	3.1%
Profit and ROI	4.5%	3.6%
Eligible Importers	4.3%	0.3%
Procurement	3.1%	5.3%
Litigation	2.5%	2.1%
CAMR Outcomes and uptake	0.0%	21.0%
**Security**		
Affordability	8.3%	12.1%
Development	7.5%	11.9%
Quality and Safety	2.3%	3.5%
Innovation	2.0%	1.6%
Human Rights	1.4%	0.4%
Domestic Economy	0.1%	0.6%
Neglected Diseases	0.3%	0.3%

^1 ^In 2004, the denominator = 108536 words (i.e. total number of words in ALL 2004 debates)

^2 ^These percentages aggregate all stakeholders and political parties. Percentages represent proportion of text where specified themes were found. Paragraphs often made reference to more than one concept; therefore paragraphs were often coded for multiple themes.

^3 ^In 2007, the denominator = 55530 words (i.e. total number of words in ALL 2007 debates)

**Table 4:** Proportion of debates coded by theme by stakeholder^1, 2, 3^

			**2004**				**2007**		
		
	**Research Industry**	**Generic Industry**	**Civil Society**	**Government**	**IPIC**	**Research Industry**	**Generic Industry**	**Civil Society**	**Government**
	
	**(n = 3622)**	**(n = 2938)**	**(n = 31610)**	**(n = 19956)**	**(n = 3327)**	**(n = 8785)**	**(n = 4146)**	**(n = 14327)**	**(n = 11330)**
**Equity**									
Aid	41%	25%	17%	13%	0%	29%	12%	25%	29%
List of Countries	1%	0%	7%	11%	0%	0%	0%	0%	6%
List of Medicines	0%	4%	12%	18%	0%	3%	2%	8%	3%
Equal Opportunity to supply	22%	1%	2%	0%	0%	0%	0%	1%	0%
**Security**									
Drug Affordability	12%	19%	15%	2%	19%	17%	21%	17%	7%
Development	8%	0%	17%	5%	0%	19%	2%	11%	12%
Domestic Economy	0%	4%	0%	0%	0%	1%	4%	0%	0%
Human Rights	0%	0%	4%	0%	0%	0%	0%	1%	1%
Innovation	2%	1%	2%	4%	0%	2%	5%	2%	1%
Quality and Safety	3%	9%	1%	5%	4%	2%	4%	3%	10%
Neglected Diseases						0%	0%	1%	0%
**Liberty**									
Intellectual Property	23%	44%	25%	41%	63%	34%	43%	23%	37%
Developing Country Pressure	0%	0%	2%	0%	0%	0%	4%	5%	0%
Right of Refusal	10%	8%	16%	7%	18%	0%	0%	0%	0%
WTO or TRIPS	21%	13%	27%	22%	11%	12%	7%	23%	21%
**Efficiency**									
Diversion	13%	0%	2%	10%	9%	5%	3%	2%	2%
Litigation	0%	0%	0%	7%	6%	0%	1%	4%	3%
Market Competition	4%	13%	11%	1%	11%	1%	11%	4%	1%
Procurement	15%	6%	4%	2%	5%	5%	3%	10%	3%
Profit and ROI	3%	7%	4%	6%	4%	4%	6%	3%	3%
Eligible Importers	0%	14%	6%	3%	0%	0%	0%	1%	0%
CAMR Outcomes and Uptake	0%	0%	0%	0%	0%	18%	13%	14%	24%

^1 ^n = total number of words per testimony of stakeholder group. Unit of analysis = stakeholder-testimony.

^2 ^Percentages represent proportion of text where specified themes were found. Paragraphs often made reference to more than one concept; therefore paragraphs were often coded for multiple themes.

^3 ^IPIC stands for the Intellectual Property Institute of Canada.

**Table 5:** Proportion of debates coded by theme by political party (MPs) 1,2

	**2004**				**2007**				
		
	**Liberals**	**Conservatives**	**Bloc Quebecois**	**NDP**	**Liberals**	**Conservatives**	**Bloc Quebecois**	**NDP**	**Independent**
		
	**(n = 25593)**	**(n = 7864)**	**(n = 5467)**	**(n = 8159)**	**(n = 5474)**	**(n = 4935)**	**(n = 2013)**	**(n = 3168)**	**(n = 1352)**
**Equity**									
Aid	3%	8%	6%	6%	33%	11%	22%	2%	9%
List of Countries	18%	14%	10%	11%	1%	2%	0%	3%	0%
List of Medicines	12%	14%	8%	16%	0%	7%	19%	4%	0%
Equal Opportunity to supply	2%	0%	6%	0%	0%	0%	0%	0%	0%
**Security**									
Drug Affordability	6%	1%	0%	7%	11%	2%	10%	6%	0%
Development	2%	5%	4%	4%	6%	9%	24%	7%	24%
Domestic Economy	0%	0%	0%	0%	0%	1%	0%	0%	0%
Human Rights	1%	0%	0%	0%	0%	0%	0%	0%	0%
Innovation	2%	3%	0%	0%	0%	2%	0%	0%	4%
Quality and Safety	1%	1%	0%	2%	0%	2%	0%	0%	0%
**Liberty**									
Intellectual Property	36%	22%	20%	25%	17%	29%	20%	11%	7%
Developing Country Pressure	0%	0%	0%	0%	2%	0%	0%	4%	0%
Right of Refusal	10%	9%	2%	8%	0%	0%	0%	0%	0%
WTO or TRIPS	15%	5%	8%	10%	11%	2%	6%	4%	20%
**Efficiency**									
Diversion	7%	5%	14%	1%	1%	0%	10%	0%	14%
Litigation	1%	4%	7%	2%	1%	3%	0%	0%	0%
Market Competition	4%	0%	0%	0%	3%	5%	0%	0%	0%
Procurement	1%	3%	1%	4%	8%	0%	5%	0%	3%
Profit and ROI	7%	0%	0%	3%	5%	6%	2%	4%	0%
Eligible Importers	3%	4%	11%	3%	0%	1%	0%	0%	0%
CAMR Outcomes and Uptake	0%	0%	0%	0%	21%	16%	33%	49%	41%

^1 ^n = total number of words per testimony of stakeholder group. Unit of analysis = stakeholder-testimony.

^2 ^Percentages represent proportion of text where specified themes were found. Paragraphs often made reference to more than one concept; therefore paragraphs were often coded for multiple themes.

Overall, these statistics back up the three main findings outlined in this paper. First, the combination of participants' focus on intellectual property and TRIPS along with the institutional context in which this debate took place led the discourse in a certain direction. By framing the initiative as the implementation of the Paragraph 6 Decision, the Liberal government immediately channelled the debate into the domestic institutions that had authority over pharmaceutical patent law, along with their policy legacy, preferences, norms, and rules. Since the venue largely dictates how an issue will be framed and can influence how actors perceive and articulate their interests, intellectual property and the WTO TRIPS Agreement became the terms of debate for most stakeholders [16,36]. Given that the goal of protecting intellectual property, as promoted by the research-based industry, was entrenched in the bureaucracy, Canada's economic structure, international trade rules and electoral considerations of the most politicians on the Standing Committee (except for the NDP), these goals ended up taking priority over most other considerations, including the goals of drug affordability (8.3%) through market competition (5.1%). These institutional factors appeared to have the largest impact on what the final design of CAMR would look like.

Second, the goals or issues that received less attention during these debates were later identified in 2007 as major barriers to the use of the legislation by civil society and the generic industry.^p ^These include CAMR's lack of congruence with developing country procurement practices (3.1%), the threat of litigation against participating generic companies (2.5%) and political or economic pressure placed on developing countries by more powerful countries to avoid using TRIPS flexibilities altogether (developing country pressure, 0.5%).

Finally, issues that were hardly discussed included: innovation (2.0%), human rights (1.4%) and the Canadian domestic economy (0.1%). As this paper argues, the initial problem definition played a role in deemphasizing these issues; however, the receptivity of the leading institutions to these frames also affected the scope of issues up for debate. In particular, three relationships were largely overlooked during these debates: 1) the relationship between intellectual property protection, TRIPS and innovation; 2) the impact of CAMR on the Canadian domestic economy; and, 3) the relationship of human rights in relation to drug access, patents and TRIPS. For example, the virtually negligible role that human rights played throughout these hearings, points to the lack of receptivity of the policy venue to that policy goal.

As seen in Table 2, the largest increase observed in themes moving from 2004 to 2007 is in the frequency of efficiency goals (an increase of 12% in the proportion of text coded as efficiency). This represents the amount of time spent discussing CAMR's outcomes, implementation and uptake by developing countries and the Canadian generic industry (Tables 4 and 5). This is not surprising as the debates themselves were framed specifically for this purpose: to review the legislation, three years on, to assess whether any amendments had to be made to the regime.

The next change observed is a decrease in references to liberty goals (decrease of 10%). This observation can be explained by the focus of most participants in 2004 on the right of refusal (10.7%), which was no longer an issue in 2007 (0%). Instead, there was a greater emphasis by participants in exploring other ways to improve drug affordability (increase in 3.8%), especially through aid programs (increase in 11.6%). The emphasis on aid likely signals a shift towards pursuing voluntary policy tools, such as corporate philanthropy and voluntary price reductions, and a movement away from compulsory licensing to pursue affordable drug access.

## Discussion

Through a systematic analysis of the framing of policy debates, stakeholder interests and the relevant institutions, this analysis aimed to determine how stakeholders framed access to medicines in the case of CAMR and how framing, institutions and interests led to the policy product known today as Canada's Access to Medicines Regime. In 2004, policy debates were dominated by two themes overall: intellectual property rights and TRIPS compliance. Promoting the right to health through access to essential medicines and the impact of CAMR on innovation was hardly discussed. With the Departments of Industry Canada and International Trade as the lead institutions, the goals of protecting intellectual property and ensuring good trade relations with the United States appear to have taken priority over encouraging generic competition to achieve drug affordability. The result was a more limited interpretation of patent flexibilities under the WTO Paragraph 6 Decision. Perhaps the most striking finding is the minimal discussion over the potential barriers developing country beneficiaries might face when attempting to use compulsory licensing, including their reluctance to use TRIPS flexibilities, constraints inherent in the WTO Paragraph 6 Decision and reconciling many developing countries' desire to pursue technological development. Instead, these issues were raised in 2007, which can be partly accounted for by a greater representation of the interests of some developing country governments.

The 2004 content analysis results confirm the findings in similar studies looking at TRIPS-related policy debates over access to medicines, to show that intellectual property issues dominated the policy contents [37-39]. In particular, the focus on protecting intellectual property, complying with the TRIPS Agreement and the right of refusal clause effectively displaced the deliberation of other equally valid policy goals as well as upstream policy implementation issues. Further confirming findings from prior literature, the underlying interests within this debate appeared to be divided into two positions: those who advocated a more limited use of compulsory licensing and those who wanted a broader, more flexible regime. Not surprisingly, the research-based industry, backed by the Intellectual Property Institute of Canada, was in the former and civil society was in the latter. Meanwhile, the generic industry appeared to hold a mixed position throughout the debates. In 2004, they seemed like reluctant participants, given the lack of commercial incentive permitted by the Paragraph 6 Decision. After Apotex got involved in CAMR's implementation, the generic industry may have had more vested interests in the outcome of the 2007 debates, and held a position in line with civil society, advocating for a streamlined and easy-to-use compulsory licensing regime.

Overall, the 2004 Canadian debates occurred within a limited policy space to start with. By framing the problem as the WTO Paragraph 6 Decision, the Liberal Government already influenced the direction of the policy process. The Liberal Government effectively imported its policy positions from the WTO negotiations, many of which developing countries and civil society protested at the time. In the Canadian context, the result was a legislation that largely favoured protecting intellectual property and a more limited interpretation of TRIPS when it comes to permitting patent flexibilities.

Of further importance is the private and non-transparent process by which the first draft of the legislation got designed - private consultations that occurred prior to the public hearings. The only participants in these private consultations were representatives of the research-based industry, the generic industry and civil society. As Howlett describes, this process itself is quite common as it allows for the Government to control the scope of the policy debate;[36] however, it was during this stage that the legislation became quickly grounded in a more restricted interpretation of TRIPS, having reintroduced limitations on the scope of medicines and countries, as well as outlining specific restrictions on the nature of compulsory license under the regime. The public debates that permitted closer scrutiny, were conflicts over what was already a very narrow range of alternatives to choose from.

The most significant factor in the outcome of the 2007 debates appears to be the preference of the Conservative Government to maintain the status quo and leave CAMR unchanged. The Conservative government had little incentive to improve the legislation since it was a Liberal initiative. Moreover, under their new leadership, Canada's foreign policy took on a more aggressive approach with respect to intellectual property protection.^r ^For example, the Department of Foreign Affairs and International Trade (DFAIT) announced in 2007 that it was assessing its interests in protecting intellectual property as it initiated trade agreements in Peru, Colombia and the Dominican Republic.^s ^The Government's stated intent was to protect the interests of Canadian firms and intellectual property owners in foreign markets given the increased importance of intellectual property in Canada's knowledge-based economy. This new policy position made the prospects for any amendments to the regime unlikely from the start.

The qualitative contents of the policy debates in 2007 showed a much larger range of issues up for discussion when compared with 2004. Debates focused largely on the efficiency of CAMR, identifying intellectual property protection and upstream implementation issues as impeding the participation of both the generic industry and developing country governments. The framing differences observed may partly be explained by the nature of the policy process: the focus on one issue naturally comes at the expense of another [40]. It appears as though in 2004, civil society and the generic industry's focus on the right of refusal, the lists of medicines, the list of countries and allowing NGOs to be eligible purchasers under the regime, came at a cost of lobbying for other changes to the legislation.

One barrier received significant attention in the 2007 debates but failed to resonate when briefly mentioned in 2004. Developing country pressure was mentioned by civil society in 2004 but it was not discussed by any other stakeholder or politician until 2007, when most stakeholders and politicians acknowledged that country notification was a major impediment to CAMR's success.

Neither civil society nor the generic industry was concerned with country notification, profit limits and delays in voluntary license negotiations in the 2004. Their silence was likely because the policy alternatives they were facing were much worse; however, that these issues are rooted in the WTO Paragraph 6 Decision itself leads to the obvious question of why civil society and the generic industry lobbied for the implementation of the Paragraph 6 Decision in Canada, despite their opinion that the Decision was flawed [41]. It is possible that they have an incremental policy approach in mind, as they are now calling on more fundamental changes to CAMR and the WTO system in general [36]. Most recently, Canadian civil society led a new campaign to introduce legislation to amend CAMR to facilitate a streamlined, "one-license solution" [42]. While the Canadian Parliament passed this solution under Bill C-393, it was then blocked the Conservative majority in the Canadian Senate. Given the new Conservative majority government recently elected in Canada, future reform of CAMR seems highly unlikely.

The framing differences observed between 2007 and 2004 can also be partly attributed to a greater representation of the interests of developing country governments. While the range of stakeholders involved in the 2007 legislative review remained similar to 2004, civil society, the generic industry, government civil servants and even the research-based industry, had more interaction with beneficiary country representatives which was specific to CAMR, yielding new and more relevant information. Hence, a much larger range of issues came to the fore. Had representatives of developing country governments been more involved during the 2004 consultations, it is possible that these issues may have become more prominently discussed or considered.

Both sets of debates saw a number of issues pertinent to drug access not raised, or if they were, they were not taken up by the political system. These under-represented themes included innovation, the domestic economy and human rights. The silence on these issues speaks to a larger question of the assumptions underlying policy debates on access to medicines and intellectual property and policy alternatives that were not explored.

Focusing on human rights in particular, it appears as though both civil society and the Liberal Government mentioned the issue of human rights but it did not have much resonance within the political system. Civil society's silence on the issue of human rights was likely a strategic decision to frame their discourse to suit the Standing Committee's needs.^t ^Perhaps more striking is the contrast between the Liberal and Conservative Government's discourse on human rights, which appear to be rooted in a very different conception of the realization of the right to health. The Liberal government framed CAMR early on as a tool to pursue human rights and a Liberal MP further suggested that Canada's responsibility includes running projects to help implement CAMR in other countries [43,44]^u^. These goals did not get incorporated into the final policy design. In contrast, the Conservative Government did not view their responsibilities as extending to the promotion of the right to health for those in other countries.^v ^Underlying these contrasting views leads to a much broader question, which is: what are states' obligations to ensuring access to medicines in other countries and how does this relate to the WTO Paragraph 6 Decision.

From the perspective of the public policy process, the case study and analysis presented in this study shows that framing can obscure the real trade-offs in a policy debates. For the research-based industry and its supporters, compulsory licensing is framed as breaking patents, theft and piracy. Throughout policy debates in many areas, social conservatives' arguments against government intervention share a similar connotation: government intervention is labelled as impinging on individual liberty and as inherently inefficient [13]. This widely held idea prevents many innovative policy balances from being achieved. Evidence suggests that the use of compulsory licensing in countries where there is negligible pharmaceutical purchasing power will not be detrimental to pharmaceutical innovation [45]. But those strongly opposed to the use of compulsory licensing in developing countries appear to be more concerned about a slippery slope; a fear that compulsory licensing would become a more widely used policy tool and eventually cut into the markets and R&D budgets of the research-based industry. As a consequence, the gradual decline of compulsory licensing as a legitimate policy tool to address drug access removes one more policy tool from governments' arsenals to address the problem of high drug costs. At the time of writing, Canada remains the only country to have successfully exported medicines under the WTO Paragraph 6 mechanism and the HIV/AIDS medicines order to Rwanda remains the only instance where CAMR has been used.

### Global drug policy developments: does Paragraph 6 still matter?

From a larger global drug policy perspective, there has been a large increase in international efforts aimed at improving drug access, including the growth of the Global Fund to fight AIDS, Tuberculosis and Malaria (GFATM), UNITAID and the Clinton Health Access Initiative (CHAI). Since the Global Fund was established in 2002, it has put 3 million people on antiretroviral therapy worldwide.^w ^UNITAID, an international financing facility started by Brazil, Chile, France, Norway and the United Kingdom in 2006, has raised as much as US$1.3 billion mainly through airline levies and contributions from the Gates Foundation.^x ^By providing the financing necessary and collaborating with the Clinton Health Access Initiative's "forward pricing" mechanisms, UNITAID helped bring the price of paediatric medicines down by 64% and tenofovir by more than 70%.^y^

The Medicines Patent Pool, which transitioned out of UNITAID in November 2010, involves cooperation with the research-based industry around the voluntary licensing for certain drugs (ARVs).^z ^In September 2010, it secured its first license from the US National Institutes of Health, which covers a series of patents related to darunavir.^aa ^In 2011, Gilead reached an agreement, which allows for the production of tenofovir, emtricitabine, cobicistat, and elvitegravir as well as a combination of these products.^ab ^However, the success of the patent pool rests solely on companies' willingness to donate their patents voluntarily on a case by case. Most recently, Johnson and Johnson refused to donate its patents on rilpivirine, darunavir, and etravirine.^ac ^Companies appear to be more interested in older ARVs and they are asking for market segmentation - middle-income countries like Brazil, India, South Africa and China may be prevented from either benefiting or participating in the patent pool.^ad^

Despite these developments in drug affordability and access, current options for second and third line antiretrovirals are still limited. Orsi and d'Almeida report that although first-line therapy has benefited from market competition, second and third line antiretroviral drugs run significantly higher at rates such as US$ 610 and 1660 per patient per year [46]. Prices in middle-income countries can run significantly two to three times higher [46]. It is from this perspective that global civil society activists still promote a role for compulsory licensing and the Paragraph 6 Decision, as a stick that country governments could use, to either speed up the process of obtaining these price reductions in needed areas of treatment, or to address those medicines where patent-holders are not willing to cooperate. Some developing countries, including India, argue that the Canadian experience shows that the Decision is not working [47]^ae^. Clearly, some developing countries still believe that Paragraph 6 matters but CAMR's potential role in improving global drug access is still unclear. There may be potential for Canada to take advantage of its sophisticated manufacturing capacity through the production and export of active pharmaceutical ingredients or other expensive and difficult to produce products.^af ^Given Canada's sophisticated generic manufacturing capacity, CAMR may be able to serve a specialized role but the overall magnitude of CAMR's contribution to improving global drug access will likely be small.

### Limitations

There are limitations in our research. First, within the range of policies that exist to address access to medicines, the case of CAMR is a narrow and specific one. A broader case study could provide more information on the range of possible policy alternatives that are available to pursue drug access and to better assess how institutions and interests favour some policy alternatives over others. This case was chosen, however, due to its urgency as a policy dilemma and as a critical global drug policy question: what political factors influence policies that aim to reduce drug prices through the use of patent flexibilities? Furthermore, given CAMR's relevance to the global Paragraph 6 debate--Canada remains the only country to have produced and exported under the Paragraph 6 mechanism--the findings from this research have global drug policy significance. Specifically, CAMR has important lessons to pass on to civil society advocates and policy-makers worldwide about the conditions under which such flexibilities may or may not be politically feasible. The findings from this study can inform advocates' and policy-makers' framing strategies, their use of institutions and scope of conflict, to inform future policy initiatives in this area.

The mixed method content analysis technique imposed its own limitations on the research but these limits are highly related to the heterogeneity of the literature in which this study is based. This thesis research appears to be the only rigorous, comprehensive analysis of policy contents in the area of drug access and intellectual property. The use of a codebook in this study was required to ensure reliability and validity of the findings. That said, the codebook is unique therefore the transferability of the quantitative content analysis results are limited to the themes searched for. However, the qualitative summaries of the themes provide sufficient information to the reader who may be seek a different perspective on how stakeholders framed the issues.

Lastly, the content analysis was limited to the standing committee hearing transcripts. These data boundaries were drawn in relation to ensuring a representative cross-section of the debates were used and also out of respect of the resource and time limitations of the research itself. Of course, a wider swath of data over a larger period of time likely would have covered more themes and more actors. Nevertheless, the case boundaries were defined much more broadly, with the literature review covering documents prior to the WTO Paragraph 6 Decision and up until the 2007 debates. Furthermore, this study included discussion of issues up until mid-2011 in relation to the developments around CAMR, Paragraph 6, and developments globally on drug access. This context helped triangulate the findings of the content analysis and provided additional data to ensure a complete picture of CAMR's political development was presented.

## Conclusions

The Canadian government tried to balance between drug affordability and intellectual property protection but it designed CAMR as a last resort measure. Increased input from the developing country beneficiaries and shifting to institutions where the right to health gets prioritized may lead to policies that better achieves affordable drug access.

## Competing interests

LCE provided research support on Canada's Access to Medicines Regime while working as an unpaid intern for Médecins Sans Frontières' (MSF) in 2005 and as a paid consultant with the Canadian HIV/AIDS Legal Network and the North South Institute in April 2007.

## Authors' contributions

LCE conceived and designed the study, analyzed and interpreted the data, and drafted the manuscript. JCK contributed to the design of the study, interpretation, and critically revised the manuscript for important intellectual content. All authors have given final approval of the version to be published.

## Endnotes

^a^The Global Fund has placed more than 3 million people on antiretroviral therapy since 2002 and the Gates Foundation, who has donated more than US$2.2 billion in HIV grants to organizations around the world. From: Overview: The Gates Foundation's HIV Strategy, July 2010. http://www.gatesfoundation.org/hivaids/Documents/hiv-strategy-overview.pdf [Date Accessed: 26 June 2011].

^b^UNAIDS estimates that approximately 170,000 people were living with HIV in Rwanda in 2009. UNAIDS: Rwanda. Available at: http://www.unaids.org/en/regionscountries/countries/rwanda/ [Date accessed: 22 January 2012].

^c^Obviously, numerous other values can be and are a part of policy debates; however, these four ideas are useful for understanding the trade-offs that governments face between policy goals.

^d^Provisions also exist to prevent or remedy anti-competitive practice (Articles 8.2, 31(k) and 40). Article 30 permits an early working provision, which allows generic companies to obtain product approval and enter the market immediately upon patent expiration.

^e^Lexchin, J. Brief to the Industry, Science and Technology Committee on Bill C-9, An Act to Amend the Patent Act and the Food and Drugs Act. February 23, 2004.

^f^The research-based pharmaceutical industry is generally opposed to the routine use of compulsory licensing based upon its potential for several harmful effects, arguing that compulsory licensing: 1) reduces prospects for economic growth in developing countries that adopt it; 2) kills the incentives for pharmaceutical firms to innovate and to introduce new products into the country; 3) encourages free-riding by imitator firms and denies local firms the opportunity to participate in technology transfer in these countries; and 4) generally demonstrates "a lack of respect for intellectual property rights,". [Rozek RP. The effects of compulsory licensing on innovation and access to health care. The Journal of World Intellectual Property. 2000;3(6):889-917]. Given these potential consequences, the research-based industry and its supporters oppose "broad-based compulsory licensing" arguing that compulsory licensing, as stipulated by Article 31 of the TRIPS Agreement, is intended to be of limited use, mainly to address anti-trust issues. Historically, compulsory licenses have been more frequently granted as judicial remedies for violation of competition laws. See: "Examples of Health-related Compulsory Licenses" at: http://www.cptech.org/ip/health/cl/recent-examples.html

^g^Compulsory licensing to manufacture was a part of the Patent Act since 1923.

^h^The legislative initiative was first tabled in Parliament as Bill C-56 and then reintroduced in the following session as Bill C-9.

^i^Bill C-56, An Act to amend the Patent Act and the Food and Drugs Act, 2nd Sess., 36^th ^Parl., 2003 (1^st ^reading 6 November 2003). The legislation was passed as the "Jean Chretien Pledge to Africa Act", and was subsequently renamed by the new Conservative Government, "Canada's Access to Medicines Regime."

^j^The information includes: "the name of the licensed product, as set out in Schedule 1, and, if applicable, the strength, dosage form and route of administration; its distinguishing characteristics; the identity of the importing country; the amount to be manufactured and sold for export; information identifying every known party who will be handling the product while it is in transit from Canada to the importing country; and the export tracking number and number of the bill of lading for each shipment."

^k^This is referred to as the "Good Faith Clause". Patent holders can challenge a compulsory licence if they can prove that "the average price of the licensed drug or medical device is 25 percent or more of the average price of the equivalent patented product in Canada. The licence holder has an absolute defence if the licence holder can establish that the average price of the drug or medical device remains less than its direct supply cost, plus 15 percent."

^l^Additional grounds under which a patent-holder can challenge the license include: if any information in the application is inaccurate; if the license holder fails to meet the conditions of the license including: "establishing and maintaining a website; providing for all shipments an export notice to the patent holder, the importing country and the purchaser; paying the prescribed royalty to the patent holder; or, providing the patent holder and the Commissioner of Patents with a copy of any supply agreement related to the licence."; if the exported product exceeded the specified quantity; "if the product was used by a non-WTO member country for commercial purposes; or if the country failed to adopt anti-diversion measures as specified by Article 4 of the Decision."

^m^See: QSR International, Available at: http://www.qsrinternational.com

^n^The chair only votes unless there is a tie. http://www2.parl.gc.ca/CommitteeBusiness/CommitteeMembership.aspx?Cmte=INST&Language=E&Mode=1&Parl=37&Ses=3 [accessed: 6 September 2009]

^o^The research-based industry was represented by the President of Canada's Research-Based Pharmaceutical Companies (Rx&D), and one representative of Eli Lilly and Company. The Canadian Generic Pharmaceutical Association was represented by its President, and one industry executive from Novopharm Limited.

^p^The barriers to CAMR's success are largely dependent upon whose perspective is assumed, which is one of the findings of this study. For the purposes of this paper, I have used civil society and the generic industry's concerns as a reference point for the barriers to CAMR's use based upon the premise that they are the key stakeholders in the implementation of the regime. These barriers were based on their positions during the 2007 hearings, and triangulated by information from their 2007 consultation submissions and the Standing Committee's final report to the Government.

^q^Other barriers that civil society and the generic industry identified but will be discussed later include delays associated with voluntary license negotiations, restrictions on the duration, quantity and number of countries per compulsory license, and perhaps most importantly, the requirements of the WTO Paragraph 6 Decision itself.

^r^Letter from Richard Elliott (Canadian HIV/AIDS Legal Network) to Lesia Stangret, Assistant Deputy Director, Intellectual Property, Information and Technology Trade Policy Division, Department of Foreign Affairs and International Trade, Re: Intellectual Property issues in bilateral free trade agreements. July 17, 2007.

^s^"Subjects For Closed Consultations: Intellectual Property. The Government of Canada is seeking the Views of Canadians to Assess Canadian Intellectual Property Interests in Selected Markets". Date accessed: 12 March 2010. From: From: http://www.international.gc.ca/consultations/closed-anterieures2.aspx?lang=en

^t^For example, an entire section of the Canadian HIV/AIDS Legal Network's written submission to the Standing Committee framed a less restrictive interpretation of the WTO Paragraph 6 Decision in terms of Canada's international human rights obligations.

^u^The Government's initial discourse in the media framed CAMR as a tool to achieve human rights: "The current initiative sets a unique global standard on the frontiers of public health and human rights."(See Graham, 2003)

^v^The Conservative Government stated, "...While that covenant [the International Covenant on Economic, Social and Cultural Rights] requires each state party to promote the right to health for its own citizens, there is no interstate obligation to protect the right in other countries, and while all international development assistance, including health-related assistance, is a moral and not a legal obligation, Canada has been a major donor to health-related initiatives in the developing world."

^w^The Global Fund to Fight AIDS, Tuberculosis and Malaria. "Fighting AIDS, Tuberculosis and Malaria." Available at: <http://www.theglobalfund.org/en/about/diseases/> [Date Accessed: 26 June 2011].

^x^See UNITAID webpage. Available at: http://www.unitaid.eu/who/background?id=159 [Accessed 10 July 2010].

^y^"How UNITAID Works in Markets". May 2011. Available at: http://www.unitaid.eu/images/Factsheets/md_factsheet_2011_en.pdf [Date Accessed: 26 June 2011].

^z^Medicines Patent Pool. Home Page. Available at: <http://www.medicinespatentpool.org/> [Date accessed: 26 June 2011]

^aa^Medicines Patent Pool. "Current Licenses". Available at: http://www.medicinespatentpool.org/LICENSING/Current-Licences [Date accessed: 26 June 2011].

^ab^Medicines Patent Pool. "Medicines Patent Pool Signs Licences to Increase Access to HIV/AIDS Medicines." Press release. 12 July 2011. Available at: http://www.medicinespatentpool.org/medicines-patent-pool-announces-first-licensing-agreement-with-a-pharmaceutical-company/ [Date accessed 23 January 2012].

^ac^MSF Access to Medicines Campaign. 2011. "Johnson & Johnson Turns Its Back on AIDS Patients." 25 April 2011. Available at: <http://www.msfaccess.org/about-us/media-room/press-releases/johnson-johnson-turns-its-back-aids-patients> [Date accessed: 26 June 2011].

^ad^See" Make It Happen", MSF Campaign for Access to Essential Medicines, November 30, 2009. Available at: http://www.msfaccess.org/make-it-happen [Accessed 10 July 2010].

^ae^See also, "Members ask: is the "Par.6" system on intellectual property and health working?" WTO webpage, available at: http://www.wto.org/english/news_e/news10_e/trip_02mar10_e.htm [Accessed 10 July 2010].

^af^As argued by civil society representatives at the 2007 debates. See: Canada. Parliament. House of Commons. Standing Committee on Industry, Science and Technology. Evidence. (April 16, 2007) 39th Parliament, 1st Session.

## Supplementary Material

Additional file 1 **Codebook for content analysis**. Click here for file

## References

[B1] World Health Organization The World Medicines Situation 2004 Geneva: World Health Organization

[B2] Frost LJ Reich MR Access: how do good health technologies get to poor people in poor countries 2008 Cambridge, MA: Harvard University Press

[B3] World Health Organization How to develop and implement a national drug policy 2003 6 Geneva. WHO

[B4] UNAIDS UNAIDS Report on the Global AIDS Epidemic 2010 Geneva: Joint United Nations Programme on HIV/AIDS (UNAIDS)

[B5] Mendis S Fukino K Cameron A Laing R Filipe A Jr Khatib O The availability and affordability of selected essential medicines for chronic diseases in six low-and middle-income countries Bulletin of the World Health Organization 2007 85 279 288 10.2471/BLT.06.033647 17546309 PMC2636320

[B6] Kohler JC Reflections on pills and poverty CPJ 2007 140 379

[B7] World Trade Organization Implementation of Paragraph 6 of the Doha Declaration on the TRIPS Agreement and Public Health: Decision of the General Council of 30 August 2003 http://www.wto.org/english/tratop_e/trips_e/implem_para6_e.htm

[B8] Canada's Access to Medicines Regime http://www.camr-rcam.gc.ca/index_e.html

[B9] Attaran A A tragically naive Canadian law for tragically neglected global health Canadian Medical Association Journal 2007 176 1726 1727 10.1503/cmaj.070409 17449707 PMC1877845

[B10] Cohen-Kohler JC Esmail LC Cosio AP Canada's implementation of the Paragraph 6 Decision: Is it sustainable public policy? Globalization and Health 2007 3 12 10.1186/1744-8603-3-12 18062821 PMC2180169

[B11] Elliott R Delivery past due: global precedent set under Canada's Access to Medicines Regime HIV/AIDS Policy & Law Review/Canadian HIV/AIDS Legal Network 2008 13 1 5 18724448

[B12] Schön DA Rein M Frame reflection: Toward the resolution of intractable policy controversies 1994 New York: Basic Books

[B13] Stone DA Policy paradox: The art of political decision making 2002 2 New York: Norton

[B14] Krasner SD International regimes 1983 Ithaca: Cornell University Press

[B15] Schattschneider EE The semisovereign people: a realist's view of democracy in America 1964 New York: Holt, Rinehart and Winston

[B16] Baumgartner FR Jones BD Agendas and instability in American politics 1993 Chicago: University of Chicago Press

[B17] World Trade Organization Fact sheet: TRIPS and pharmaceutical patents. obligations and exceptions http://www.wto.org/english/tratop_E/trips_e/factsheet_pharm02_e.htm

[B18] Correa CM Implications of the Doha Declaration on the TRIPS Agreement and public health 2002 Geneva: World Health Organization, Essential Drugs and Medicine Policy

[B19] Médecins sans Frontières Examples of the importance of India as the "pharmacy for the developing world" 2007 Geneva: Médecins sans Frontières

[B20] Outterson K Should access to medicines and TRIPS flexibilities be limited to specific diseases? American Journal of Law and Medicine 2008 34 279 301 18697695 10.1177/009885880803400208

[B21] World Trade Organization Doha WTO Ministerial 2001: Declaration on the TRIPS agreement and public health, adopted on 14 November 2001 http://www.wto.org/english/thewto_e/minist_e/min01_e/mindecl_trips_e.htm

[B22] Lexchin J After compulsory licensing: coming issues in Canadian pharmaceutical policy and politics Health Policy 1997 40 69 80 10.1016/S0168-8510(96)00886-X 10165902

[B23] Jenish D Trials and triumphs: The remarkable story of Canada's generic pharmaceutical industry 2003 Canada: Canadian Generic Pharmaceutical Association

[B24] Reichman J Hasenzahl C Non-voluntary licensing of patented inventions 2003 City: Geneva, ICTSD and UNCTAD Issue Paper, 5

[B25] Doern GB Sharaput M Canadian intellectual property: The politics of innovating institutions and interests 2000 Toronto: University of Toronto Press

[B26] Lexchin J Pharmaceuticals, patents, and politics: Canada and Bill C-22 International Journal of Health Services 1993 23 147 160 10.2190/UCWG-YBR3-X3L0-NWYT 8425783

[B27] Scoffield H Chase S Ottawa heeds call on AIDS 2003 Toronto: Globe and Mail A1

[B28] Elliott R Pledges and pitfalls: Canada's legislation on compulsory licensing of pharmaceuticals for export International Journal of Intellectual Property Management 2006 1 94 112 10.1504/IJIPM.2006.011024

[B29] Ng E Kohler JC Finding Flaws: The Limitations of Compulsory Licensing for Improving Access to Medicines-An International Comparison Health LJ 2008 16 143 19536980

[B30] Goodwin P Right idea, wrong result - Canada's Access to Medicines Regime American Journal of Law and Medicine 2008 34 563 580 10.1177/009885880803400404 19216248

[B31] Canadian-made life saving HIV/AIDS drug heading to Africa under Canada's Access to Medicines Regime (CAMR) http://www.apotex.com/nl/en/aboutapotex/sept23.asp

[B32] Government of Canada Report on the statutory review of sections 21.01 to 21.19 of the Patent Act 2007 Ottawa: Government of Canada

[B33] Krippendorff K Content analysis: An introduction to its methodology 2004 Thousand Oaks, CA: Sage Publications, Inc

[B34] Blackburn S The Oxford dictionary of philosophy 2005 2 Oxford: Oxford University Press

[B35] Huberman AM Miles MB Qualitative data analysis: An expanded sourcebook 1994 2 Thousand Oaks, CA: Sage Publications

[B36] Howlett M Perl A Ramesh M Studying public policy: Policy cycles & policy subsystems 2009 3 Don Mills, Ont: Oxford University Press

[B37] Abbott FM The WTO Medicines Decision: World pharmaceutical trade and the protection of public health American Journal of International Law 2005 99 317 358 10.2307/1562501

[B38] Sell SK Prakash A Using ideas strategically: The contest between business and NGO networks in intellectual property rights International Studies Quarterly 2004 48 143 175 10.1111/j.0020-8833.2004.00295.x

[B39] t'Hoen EFM The global politics of pharmaceutical monopoly power. drugs patents, access, innovation and the application of the WTO Doha Declaration on TRIPS and Public Health 2009 Diemen, The Netherlands: AMB Publishers

[B40] Kellow A Promoting elegance in policy theory: Simplifying Lowi's arenas of power Policy Studies Journal 1988 16 713 724 10.1111/j.1541-0072.1988.tb00680.x

[B41] Elliott R Canada can carry much more 2003 Toronto: Globe and Mail A23

[B42] Elliott R Fixing Access To Medicines Regime Essential 2010 http://www.countercurrents.org/elliott261010.htm

[B43] Graham B Canada leading the way 2003 Toronto: The Toronto Star A21

[B44] Martin P Speech from the throne to open the third session of the thirty-seventh Parliament of Canada 2004 Government of Canada

[B45] Commission on Intellectual Property Rights Public health, innovation and intellectual property rights: Report of the Commission on Intellectual Property Rights, Innovation and Public Health 2006 Geneva: World Health Organization

[B46] Orsi F d'Almeida C Soaring antiretroviral prices, TRIPS and TRIPS flexibilities: a burning issue for antiretroviral treatment scale-up in developing countries Current Opinion in HIV and AIDS 2010 5 237 10.1097/COH.0b013e32833860ba 20539080

[B47] Intervention of India at the WTO TRIPS Council on "Effective operation of Para 6 system" http://lists.essential.org/pipermail/ip-health/2009-March/013533.html

