# Supplementary material for: The politics behind the implementation of the WTO Paragraph 6 Decision in Canada to increase global drug access
Source: Global Health. 2012 Apr 3;8:7. doi: 10.1186/1744-8603-8-7 (PMC3388467; doi:10.1186/1744-8603-8-7)
Supplement: Additional file 1 — Codebook for content analysis. [file 1744-8603-8-7-S1.RTF]

Additional File 1: Codebook for Content Analysis
Equity Goals

Aid: discussion of any form of assistance or help given to developing countries towards improving their country, quality of life, and increasing drug access. Aid can come from countries, organizations, companies/corporations or individuals. Includes technical assistance and cooperation and 'support'. 

List of Medicines: discussion regarding the scope of drugs eligible under this legislation. "scope of drugs covered by the decision remains a subject of considerable debate…" "on the question of the scope of eligible drugs…"  

List of Countries: discussion about the list of eligible countries, otherwise known as Schedules 2, 3 and 4. These schedules restrict the eligible countries to those who are members of the WTO or are considered least-developed countries according to the UN Development Index. 

Equal Opportunity to Supply: discussion and debate about brand-name companies' counterproposal to the Right of Refusal.

Security Goals

Drug Affordability: Discussion of drug costs, expensive or cheap drug prices, or pricing policies. Keywords: low/cheap prices, expensive, costs, afford.

Development: discussion of development-related issues which are important areas that need to be addressed, or problems, barriers, or deficiencies. Development-related issues are basic facilities, services and installations needed for the functioning of a community or a society. For example: roads, schools, buildings, clean water, education. Includes technology transfer.

Canada's Domestic Economy: references to the relevance or implications of the pharmaceutical industry to the domestic economy of Canada. Examples include jobs, employment, investment.

Innovation: Innovation in general or specific to the health field, which includes discovery, research and development (R&D) of new pharmaceuticals and other medical technologies. 

Human Rights: code for discussion of human rights. As it relates to medicines, human rights may be referred to in relation to international covenants, treaties or laws.

Quality and Safety: discussion of drug quality, safety and the drug approval process. This includes references to existing global and national standards and processes. 

Liberty Goals

Intellectual Property Protection: discussion of the protection of intellectual property. Specifically focused on patents and IP as opposed to the policy tools that use/manipulate IP such as VL/CL. 

Right of Refusal: discussion and debate regarding the Right of Refusal.

Developing country pressure: references to pressure put on developing countries from other countries or institutions.

WTO TRIPS Agreement: references to the TRIPS Agreement, the Paragraph 6 Decision, the Doha Declaration and any requirements or related clauses.

Efficiency Concepts

Market competition: discussion of competition in a market between firms. Often used in reference to generic competition to lower prices.

Litigation: discussion about threats or taking legal action either against pharmaceutical companies or originating from the companies. 

Profits: discussion of profit, defined as financial gain after all investments and costs are taken into account; discussion of return on investment. Keywords: profit, "make money" "return on investment" This means profit according to: wordnet.princeton.edu/perl/webwn. 

Diversion: discussion about diversion, the situation in which pharmaceuticals that are intended for a specific market are illegally diverted to a different market and sold at a higher price. Includes reimportation and discussion of mechanisms to prevent diversion (anti-diversion) including marking and labeling. 

Procurement: discussion of pharmaceutical procurement practices, rules or laws. 

Eligible Importers: discussions of the possibility of having NGOs import the medicines, contracting directly with or purchasing the medicines directly from Canadian generic companies. 

CAMR Outcomes and Uptake: discussion about why CAMR isn't resulting in outcomes or being taken up by developing countries or the generic industry.
